# Role of advanced respiratory support in acute respiratory failure in clinically frail patients with COVID-19

**DOI:** 10.2217/fmb-2021-0226

**Published:** 2021-12-17

**Authors:** Iftikhar Nadeem, Louise Jordon, Masood Ur Rasool, Noor Mahdi, Ritesh Kumar, Zahra Rehman, Craig J Tilley, Simran Kang, Amrita Rai, She Lok, Alison McMillan

**Affiliations:** ^1^Department of Respiratory Medicine, East & North Hertfordshire NHS Trust, Stevenage, UK; ^2^Department of Respiratory Medicine, Norfolk & Norwich University Hospital NHS Trust, Norwich, UK; ^3^Department of Internal Medicine, East & North Hertfordshire NHS Trust, Stevenage, UK

**Keywords:** acute respiratory support, clinical frailty score (CFS), COVID-19, invasive mechanical ventilation, ISARIC 4C mortality score, noninvasive mechanical ventilation (CPAP & HFNO), PaO_2_/FiO_2_ Ratio

## Abstract

**Background:** The main aim of this study was to assess the efficacy of advanced respiratory support (ARS) for acute respiratory failure in do-not-attempt cardiopulmonary resuscitation order (DNACPR) COVID-19 patients. **Methods:** In this single-center study, the impact of different types of ARS modality, PaO_2_/FiO_2_ (PF) ratio, clinical frailty score (CFS) and 4C score on mortality was evaluated. **Results:** There was no significant difference in age, type of ARS modality, PF ratio and 4C scores between those who died and those who survived. Overall survival rates/hospital discharge of patients still requiring ARS at 5 and 7 days post admission were 20 and 17%, respectively. **Conclusion:** Our study showed that ARS can be a useful tool in frail, elderly and high-risk COVID-19 patients irrespective of high 4C mortality score.

Since the outbreak of COVID-19 in December 2019, the approach for managing acute respiratory failure (ARF) in COVID-19 has been under continuous review by various research and medical communities. According to initial studies, 67% of the patients admitted to intensive care developed acute respiratory distress syndrome (ARDS) [1] and that 2–17% of the patients hospitalized with COVID-19 required invasive mechanical ventilation [2–4]. Over the course of the pandemic, there has been a steady increase in the use of advanced respiratory support in ARF due to COVID-19 by around 15% as compared to ARF due to other causes (30% vs 15%) [5,6]. Advanced respiratory support (ARS) is defined as the use of non-invasive ventilation (NIV) or high-flow nasal oxygen (HFNO).

Although much of the evidence surrounding ARS use in COVID-19 has been to delay or bridge to invasive mechanical ventilation [7], it's use has been increasing in patients where escalation to invasive mechanical ventilation would not be appropriate (‘do-not-intubate’, which frequently accompanies a ‘do-not-attempt cardiopulmonary resuscitation order’; DNACPR). In practice, these decisions represent a clinical judgement based on the perceived benefit of treatment, the patient's frailty, co-morbidities and wishes. The tools that have been utilized as decision aids include the clinical frailty score (CFS) [8] and International Severe Acute Respiratory and Emerging Infection Consortium-4C Score (ISARIC 4C) [9–11]. The ISARIC 4C mortality score is a good predictor of 30-days mortality in COVID-19 (and other common respiratory tract infections).

Patients with ARF in COVID-19 where a ‘do-not-intubate’ and DNACPR decision have been made presented an important and prevalent group. A comprehensive review of available literature on the use of noninvasive ventilation in these patients showing evidence on the outcomes and predictors of mortality was scarce and suboptimal.

The main aim of this study was to evaluate the outcomes of ARS for ARF in COVID-19 patients who were not suitable candidates for invasive ventilation and had a DNACPR order in place. The secondary aims were to investigate any predictors of survival outcome such as clinical frailty score (CFS), ISARIC 4C score, age and admission PaO_2_/FiO_2_ (PF) ratio.

## Methods

### Study setting & patient selection

This was a retrospective, observational single-center study conducted at the district general hospital in UK. We included all patients who were admitted to the respiratory support unit (RSU) with ARF for treatment with ARS from 1 March 2020 to 28 February 2021 with COVID-19 pneumonia. ARS modalities included continuous positive airways pressure ventilation (CPAP), bi-level ventilation (BiPAP) or high-flow nasal oxygen (HFNO). Different ARS types were selected by clinicians depending on patient tolerability and clinical indications. Pharmacological treatment for COVID-19 was given in accordance with the latest available evidence-based national guidelines. The average time from swab positivity to admission to RSU was 11 days.

### Inclusion criteria

- Adults over the age of 18 with a valid DNACPR on admission and whose ceiling of care was ARS, excluding invasive mechanical ventilation;
- At least one positive reverse transcriptase (RT) PCR nasopharyngeal swab confirming COVID-19 infection. In addition at least one of criteria C, D and E needed to be met:
- ARS was required to treat Type 1 respiratory failure, in patients who despite maximal pharmacological and oxygen therapy were unable to maintain oxygen saturations over 92 %. Maximal oxygen therapy is defined as a flow rate of 15 l/min delivered via non-rebreathe mask or humidified FiO_2_ of 60%;
- ARS was required to treat compensated hypercapnic respiratory failure in patients who were unable to maintain oxygen saturations over 88% despite maximal pharmacological and oxygen therapy;
- ARS was required to treat decompensated hypercapnic respiratory failure.

### Exclusion criteria

- All patients who were eligible for escalation to ITU for invasive ventilation if they did not respond to ARS;
- Patients in whom ARS was discontinued prematurely due to poor compliance and tolerability. Poor tolerability was defined as anyone who was not able to use the ARS for more than 24 h or in which ARS type had to be changed from the initial modality.

### Sample size

**Figure 1.**
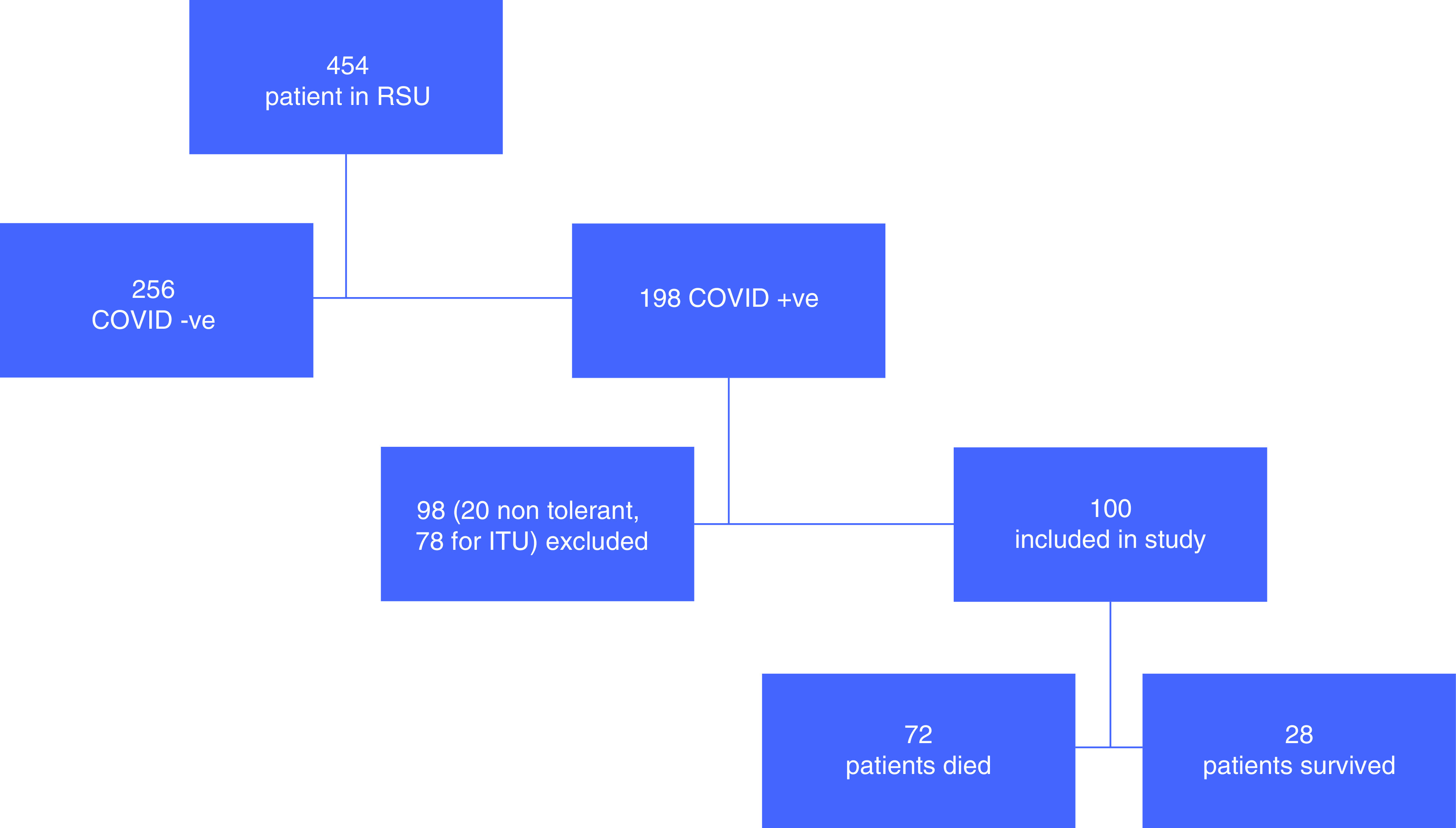
Study flow chart. ITU: Intensive therapy unit; RSU: Respiratory support unit.

A total of 454 patients were admitted to our RSU during the above time frame, 198 patients with COVID-19. 100 fulfilled the inclusion criteria. This has also been explained in flow chart (Figure 1).

### Data collection

The baseline characteristics included age, sex, clinical frailty scale, reason for DNACPR, comorbidities, date of admission, PF ratio before starting advanced respiratory support, type of advanced respiratory support, urea, respiratory rate, C-reactive protein (CRP), Glasgow coma score (GCS) and ISARIC 4C mortality score [12] at the time of initiation of advanced respiratory support. All patients' data were also included in our ICNARC data base as standard.

### Study objective

Primary outcomes included death or discharge from hospital post ARS initiation. Secondary outcomes included outcomes at 48 h, 5 days and 7 days of ARS treatment, respectively. Other secondary outcomes included the effect of ARS modality, PF ratio, CFS and ISARIC 4C score on the primary outcome.

### Statistical measure

All data analyses were performed using the JASP software package version 0.14.1. Data analysis was conducted in a pre-specified manner, and appropriate parametric and non-parametric tests were applied as per the data distribution. Where appropriate, p-values (p < 0.05) were calculated to determine significance.

### Ethical approval

In view of the observational and retrospective nature of this study, a formal ethical approval was not required as the study fell under the category of service evaluation. The data were collected as per NHS health research authority (HRA) guidance. Results are reported in accordance with the strengthening of the reporting of observational studies in epidemiology (STROBE) guidelines. Principles outlined in the Declaration of Helsinki have been followed in this study.

## Results

### Population demographics

From 1 March 2020 to 28 February 2021, 100 patients (39 females) meeting the inclusion criteria were admitted to our respiratory support unit requiring advanced respiratory support. The median age was 78 years (SEM 0.92; range 48–96 years), and median clinical frailty score (CFS) was 5 (SEM 0.09, range 3–7). The median ISARIC 4C score was 12.57 (SEM 0.29, range 2–18) which when stratified corresponded to two patients in the low-risk group, three in the intermediate-risk group, 69 in the high-risk group and 26 in the very high-risk group.

At the point of admission, 44 patients were started on high-flow nasal oxygen (HFNO), 53 on continuous positive airway pressure (CPAP) and three on BiPAP. The mean PaO_2_/FiO_2_ (PF) ratio at admission was 124.7.

### Overall outcomes

Patients were included in the study at admission to RSU and followed through until hospital discharge or death. Of the 100 patients, 72 patients died (72%) and 28 patients survived to hospital discharge (28%) (Table 1).

**Table 1.** Comparison of mean age, mean clinical frailty score, Mman PaO_2_/FiO_2_ ratio and mean international severe acute respiratory infection consortium-coronavirus clinical characterization consortium (ISARIC 4C) score on admission to the respiratory support unit, in patients who were discharged from hospital and in those who died.

Parameters	Survived (n = 28)	Died (n = 72)
Mean age (years)	75.9	76.8
Mean CFS	4.7	5.1
Mean ISARIC 4C score	12.3	12.7
Mean PF ratio at admission	126.7	123.9
ARS type (count, %): High-flow nasal oxygen (HFNO) Continuous positive airway pressure (CPAP) Bi-level positive airway pressure (BiPAP)	9 (32.1) 17 (60.7) 2 (7.2)	35 (48.6) 36 (50) 1 (1.4)

The table also shows the outcomes according to the modality of advanced respiratory support.

Table 2 describes the different co-morbidities of patients included in the study.

**Table 2.** The prevalence of co-morbidities in patients admitted to the respiratory support unit requiring advanced respiratory support for COVID-19 infection.

Co-morbidity	Patients with co-morbidity (%)
Hypertension	43
Diabetes mellitus	36
Atrial fibrillation or atrial flutter	24
Chronic obstructive pulmonary disease	24
Ischemic heart disease	21
Cancer	19
CKD	12
Hypercholesterolemia	8
Asthma	7
Congestive cardiac failure	6
Dementia	5

There was no significant difference in age between those who died and those who survived (W = 1059, p = 0.70). There was no difference in survival rates between ISARIC 4C groups (Chi square X^2^ = 6.54, df 3, p = 0.88). There was also no difference in survival rates based on the type of ARS the patient was started on (Chi square X^2^ = 3.094, df 3, p = 0.142). There was no significant difference in PF ratio at admission between those who survived and those who died (t = -0.448, p = 0.655).

When grouped by clinical frailty score, there was a significant difference in survival rates between groups (Chi square X^2^ = 11.802, df 4, p = 0.019).

Tables 3, 4 & 5 demonstrate the differences in survival rates by grouped CFS, ISARIC 4C score and ARS type (Table 3, 4 & 5).

**Table 3.** Displaying the survival rate and death rate by Dalhousie clinical frailty scale.

CFS	Status	Count	%
3	Died Survived Total	1 5 6	16.7 83.3
4	Died Survived Total	17 5 22	77.3 22.7
5	Died Survived Total	34 11 45	75.6 24.4
6	Died Survived Total	15 7 22	68.1 31.8
7	Died Survived Total	5 0 5	100 0

**Table 4.** Displaying the survival rate and death rate according to International severe acute respiratory infection consortium – coronavirus clinical characterization consortium (ISARIC 4C) score.

ISARIC 4C score	Status	Count	%
Low	Died Survived Total	0 2 2	0 100.0
Intermediate	Died Survived Total	3 0 3	100 0
High	Died Survived Total	51 18 69	73.9 26.1
Very high	Died Survived Total	18 8 26	69.2 30.8

Only three patients were present in intermediate group and 100% mortality in this group is likely due to small number. Low risk (ISARIC 4C 0–3), intermediate risk (ISARIC 4C 4–8), high risk (ISARIC 4C 9–14) and very high risk (ISARIC 4C ≥15).

**Table 5.** Displaying survival rate and death rate according to the modality of advanced respiratory support (CPAP, BIPAP and HFNO).

ARS type	Status	Count	%
High-flow nasal oxygen (HFNO)	Died Survived Total	35 9 44	79.5 20.5
Continuous positive airway pressure (CPAP)	Died Survived Total	36 17 53	67.9 32.1
Bi-level positive airway pressure (BiPAP)	Died Survived Total	1 2 3	33.3 66.7

## Predicting outcomes

Determining outcomes after a specified time on ARS is important for prognostication and relative communications. Data from the 100 patients were examined at three time points: 48 h post admission, 5 days post admission and 7 days post admission.

**Figure 2.**
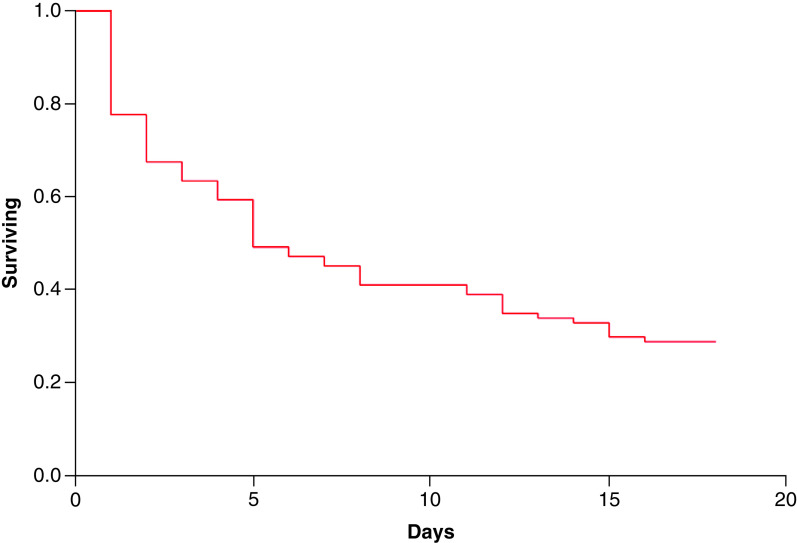
Kaplan–Meier curve to show survival to hospital discharge of study participants.

Figure 2 shows a Kaplan–Meier curve demonstrating survival to hospital discharge for the total study participants. Overall, 28% of patients survived to discharge. Data from one patient were censored, due to prolonged hospital admission.

At the 48 h time point, 21 patients had died, leaving 79 patients remaining in the cohort. When data for these 79 patients were re-assessed at 5 days, 35 patients remained on ARS (44.3%), 28 had been de-escalated to oxygen or air (35.4%) and 18 patients had died (22.8%). Of the 35 patients who were on ARS at the 5 day time point, their eventual overall outcomes were as follows: 28 patients died (80%) and seven patients survived (20%). From our cohort, overall survival rates of patients still requiring ARS at 5 days post admission is thus 20%.

When data for patients were re-assessed at 7 days, 23 patients remained on ARS (38.9%), 25 patients were stepped down to oxygen or room air (42.3%), ten patients died (16.9%) and one patient was discharged (1.7%).

Of those still requiring ARS at 7 days post admission (23 patients), their eventual overall outcomes were: 19 died (83%) and 4 survived (17%). From our cohort, overall survival rates of patients still requiring ARS at 7 days post admission is thus 17%, suggesting that if a patient still requires ARS at 7 days post admission, then their percentage chance of survival is 17% (Table 6).

**Table 6.** Displaying the percentage of patients surviving to hospital discharge after requiring ongoing advanced respiratory support (CPAP, BIPAP or HFNO) at day 5 and day 7 of admission to the respiratory support unit.

Parameters	Survival to hospital discharge rate (%)
Still requiring ARS at 5 days post admission	20%
Still requiring ARS at 7 days post admission	17%

## Discussion

In this single-center study of COVID-19 patients who were started on ARS as their ceiling of care for acute hypoxic respiratory failure, we observed that 72% died and 28% survived to hospital discharge. Of those still requiring ARS at 5 days and 7 days, the in-hospital survival rates were 20% and 17%, respectively. Patient age, PF ratio on admission, type of ARS (HFNO/CPAP/BiPAP) and ISARIC 4C score were not significantly different in those who survived and those who died, whereas increasing CFS was associated with a poorer survival rate.

These data will be of benefit in informing discussions with patients and relatives regarding prognosis when commencing ARS in this patient group.

## The benefit of ARS in ARF in COVID-19

Worldwide, the majority of patients in first wave with moderate-to-severe ARF were treated by IMV, based on criteria generally used for typical ARDS [13,14] with 49–60% mortality [15,16]. IMV is not without risks [17] and capacity is extremely limited and as such ARS has been used with increasing frequency in subsequent waves. It is not yet clear which form of ARS (if any) provides the greatest benefit. Intensive care national audit and research care's (ICNARC) data do not include patient's CFS and DNACPR status [18]. Burns GP *et al.* described a 50% survival rate (14/28 patients with median ARS duration of 5 days) which was almost same as of invasive mechanical ventilation in appropriate COVID-19 pneumonitis patients having respiratory failure [19]. In contrary, Elsheikh A *et al.* reported a 90% mortality (9/10 patients from a cohort of 45) with CPAP alone used as ceiling of care [20]. Similarly, another single-center experience stated an 83% mortality (20/24 patients with a median duration on ARS of 3 days) [21].

As seen in previous work, we observed that patients who required ARS for a longer duration (>5 days or >7 days) had significantly higher mortality [22]; potentially suggesting that ongoing ARS requirements at these time points could be an early indication of treatment failure.

## Type of ARS & mortality

We did not observe a survival difference between patients in our cohort commenced on HFNO, CPAP or BiPAP. Both HFNO and CPAP have been shown independently to reduce risk of all-cause mortality and tracheal intubation compared with supplemental oxygen alone [23], but few studies compare the two modalities, particularly in groups where ARS is the ceiling of care. A study by Brusasco *et al.* [24] showed that usefulness of CPAP in moderate to severe acute respiratory failure. Demoule *et al.* [25] revealed HFNC reduced intubation and subsequent invasive mechanical ventilation. One previous single-center RCT showed no difference in respiratory support-free days between HFNO and CPAP in COVID-19-related ARF. Procopio *et al.* [26] proposed HFNC a better treatment option than CPAP frail COVID-19 patients with respiratory failure. All these studies included patients who were for possible intubation in case the treatment with ARS failed. Notably, Recovery-RS, a global multicenter RCT, is currently recruiting to compare use of HFNC, CPAP and standard oxygen in COVID-19 patients, our center was also enrolled in this trial although it excluded patients who not suitable for invasive ventilation [27].

Possible reasons for no difference being observed between ARS types may reflect the heterogeneity in clinical and operational rationale for decisions over which modality to use e.g. concerns over hospital oxygen supply and possible heart failure element driving CPAP use and cognitive impairment favoring HFNO use.

## Clinical implications of CFS & ISARIC 4C mortality scoring

In our study patients with a premorbid frailty score of 3 were more likely to gain survival benefits from ARS compared to those with a CFS of 7. Labenz C *et al.* reported a strong positive relation between higher CFS score and the need for invasive mechanical ventilation [28]. MacMahon DP *et al.* evaluated the role of CFS scoring in predicting mortality of patients who required non-invasive ventilation due to acute hypercapnic respiratory failure in COPD/OHS/Kyphoscoliosis patients. They reported that the patients having remarkably high CFS score (7–9) had in-hospital mortality of 60% as compared to those having CFS score 1–6 (20%) [29]. Our study depicted the usefulness of CFS scoring while making treatment escalation decisions in selective patient group.

We observed a survival benefit (31%) in patients having ISARIC 4C score >14 (very high risk category) of patients who were supported by non-invasive ventilation. However, Ali R *et al.* [30] described a higher death rate (80%) without ARS support. The ISARIC 4C deterioration model developed by Gupta R K *et al.* [31] when implemented alongside ISARIC 4C mortality score has higher proportion of envisaging decline in clinical condition and hence supports in advance decision making i.e., escalating to intensive care units or non-invasive support in case of elderly and frail patients. Nevertheless, a more targeted clinical trial is required to establish the role of ISARIC 4C mortality scoring in making decisions for non-invasive ventilator support.

## Correlation with previous work, strengths & limitations

Much of the existing data surrounding ARS in COVID-19 exclude patients who are not suitable for escalation to IMV in case of treatment failure [7,32–35]. To our knowledge, this is the biggest study to date to see the clinical benefit of ARS in ARF in COVID-19 in patients who were IMV is not an appropriate treatment escalation, and to explore survival rates by ARS type, patient CFS and ISARIC 4C score. This addresses a critical gap in our current knowledge and clinical practice of COVID-19 management. Strengths of our study include the complete data set and real-world patient group, representative of many seen across the nation.

Nonetheless, our study has a few limitations. It represents admissions to a single center, a small number of patients. 95% of patients had high or very high ISARIC 4C scores, and 94% had a CFS between 4 and 7, making differentiation between these groups challenging.

Survival rates may have been lower in our cohort compared to other populations due to high CFS, multiple underlying health conditions and PF ratio showing moderate to high level of ARDS. Additionally, this was an unselected patient population and all patient treated were included.

## Conclusion

Our study showed that ARS can be a useful tool in frail, elderly and high-risk patient groups irrespective of ISARIC 4C mortality score. These findings combined with other studies will enable physicians to prognosticate in this selected patient group, to guide escalation decisions and to communicate effectively with relatives. Other measures such as patient age, admission PF ratio and ISARIC 4C score did not help to predict outcomes in this group.

On a local level, we hope this information helps guide conversations with patients and relatives. On a national and international scale, we anticipate that this work will guide larger studies to answer this question definitively.

**Summary points**

- Management of acute respiratory failure (ARF) in COVID-19 has been under continuous review by the research and medical communities.
- There has been a steady increase in the use of advanced respiratory support in ARF due to COVID-19 by around 15% as compared to ARF due to other causes (30% vs 15%).
- Majority of the times, ARS use in COVID-19 has been to delay or bridge to invasive mechanical ventilation, it's use has been increasing in patients where escalation to invasive mechanical ventilation would not be appropriate (‘do-not-intubate’, which frequently accompanies a ‘do-not-attempt cardiopulmonary resuscitation order’; DNACPR).
- Main aim of this retrospective single-center observational study was to evaluate the outcomes of ARS for ARF in COVID-19 patients who were not suitable candidates for invasive ventilation and had a DNACPR order in place. Secondary aims were to investigate any predictors of survival outcome such as clinical frailty score (CFS), ISARIC 4C score, age and admission PaO_2_/FiO_2_ (PF) ratio.
- 100 patients met the inclusion criteria. Age, sex, clinical frailty scale, PF ratio before starting advanced respiratory support, type of advanced respiratory support and ISARIC 4C mortality score at the time of initiation of advanced respiratory support were calculated.
- We found that there was no significant difference in age, type of ARS modality, PF ratio and 4C scores between those who died and those who survived. Overall survival rates/hospital discharge of patients still requiring ARS at 5 and 7 days post admission was 20 and 17% respectively.
- This study addresses a critical gap in our current knowledge and clinical practice of COVID-19 management. Our study showed that ARS can be a useful and viable tool in frail, elderly and high-risk COVID-19 patient groups irrespective of ISARIC 4C mortality score. These findings combined with other studies will enable physicians to prognosticate in this selected patient group, to guide escalation decisions and to communicate effectively with relatives. Other measures such as patient age, admission PF ratio and ISARIC 4C score did not help to predict outcomes in this group.
